# Study of High-Velocity Nasal Insufflation vs Noninvasive Positive Pressure Ventilation for Emergency Type 2 Respiratory Failure: Protocol for a Noninferiority Randomized Controlled Trial

**DOI:** 10.2196/93391

**Published:** 2026-08-14

**Authors:** Mui Teng Chua, Darius Shaw Teng Pan, Alexander Jet Yue Ng, Crystal Harn Wei Soh, Win Sen Kuan

**Affiliations:** 1 Emergency Medicine Department, National University Hospital, Singapore, Singapore; 2 Department of Surgery, Yong Loo Lin School of Medicine, National University of Singapore, Singapore, Singapore; 3 Urgent Care Centre, Alexandra Hospital, Singapore, Singapore; 4 Nuffield Department of Primary Care Health Sciences, University of Oxford, 32 Woodstock Road, Oxford, England, OX2 6GG, United Kingdom, 44 7349681776; 5 Department of Anatomy, Yong Loo Lin School of Medicine, National University of Singapore, Singapore, Singapore

**Keywords:** randomized controlled trial, respiratory acidosis, nasal oxygenation, hospital emergency service, noninvasive ventilation

## Abstract

**Background:**

Type 2 respiratory failure (T2RF) is a common and high-risk presentation in the emergency department (ED). Noninvasive positive pressure ventilation (NIPPV) is a standard first-line therapy for T2RF, particularly in acute exacerbations of chronic obstructive pulmonary disease and cardiogenic pulmonary edema. However, NIPPV has limitations, including discomfort, claustrophobia, air leaks, skin injury, and aspiration that may compromise tolerance and lead to treatment failure or escalation to endotracheal intubation. High-velocity nasal insufflation (HVNI), particularly via a single-prong asymmetric cannula configuration, has been proposed to enhance dead-space washout and improve patient comfort. Despite growing interest, robust evidence of evaluation of HVNI in heterogeneous, all-cause T2RF populations in the ED remains limited.

**Objective:**

This study aims to determine whether HVNI delivered via a single-prong nasal cannula is noninferior to standard NIPPV in improving ventilation among adult ED patients with T2RF from any cause.

**Methods:**

This is a single-center, open-label, noninferiority randomized controlled trial conducted in the ED of a tertiary academic center. Adults aged 21 years and above with T2RF, defined as partial pressure of carbon dioxide (PaCO_2_) above 45 mm Hg and pH below 7.35 requiring ventilatory support, will be randomized 1:1 to HVNI via a single-prong cannula or standard NIPPV. Allocation will be concealed via an independent web-based platform using variable block sizes. The primary outcome is percentage change in PaCO_2_ from baseline to 60 minutes after initiation of therapy. A total of 84 patients provide 80% power and one-sided α of 2.5% assuming an SD of 6.65% and noninferiority margin of 4.3%. Analyses will use intention-to-treat and per-protocol approaches, and sensitivity analyses to address missing arterial blood gas results will be conducted. Predefined failure criteria (persistent or worsening acidosis, rising PaCO_2_, refractory hypoxemia, severe intolerance, and clinical deterioration) will trigger crossover or escalation per protocol.

**Results:**

Ethics approval was granted by the local institutional ethics board on February 24, 2025 (reference 2024-4329). Recruitment commenced in January 2026 and is expected to be completed by June 2027. As of July 2026, a total of 19 participants have been enrolled out of a target sample of 84. Data analysis will commence following completion of enrollment, with results expected to be published by 2028.

**Conclusions:**

Should HVNI via a single-prong cannula be shown to be noninferior to NIPPV, it may offer a practical alternative to treatment of T2RF in the ED when mask-based interfaces are poorly tolerated or contraindicated, potentially reducing the need for more invasive interventions such as endotracheal intubation and mechanical ventilation.

## Introduction

Acute respiratory and cardiac conditions are frequently encountered in the emergency department (ED), ranging from mild to life-threatening presentations. Among patients who are critically ill, a substantial proportion present with hypercapnic, or type 2, respiratory failure (T2RF) [1]. Common etiologies include acute exacerbations of chronic obstructive pulmonary disease (COPD), pneumonia, congestive cardiac failure, restrictive lung disease, neuromuscular disease, and opioid or benzodiazepine use [2]. Early, effective ventilatory support in the ED can improve patient-centered outcomes by reducing work of breathing, correcting gas exchange, and potentially averting endotracheal intubation [3].

Noninvasive positive pressure ventilation (NIPPV) is a standard first-line treatment modality for T2RF, particularly in COPD exacerbations and cardiogenic pulmonary edema [4]. By providing inspiratory positive airway pressure (IPAP) and expiratory positive airway pressure (EPAP), NIPPV improves ventilation, reduces the work of breathing, and can correct acidosis. However, NIPPV has limitations that can affect effectiveness and tolerance, including mask-related discomfort and claustrophobia; nasal bridge skin abrasions; dry and nonhumidified air resulting in airway mucosal injury; gastric distension; aspiration; air leaks; and challenges with secretion clearance, oral intake, and communication, which may lead to therapeutic failure [5,6]. Treatment failure often necessitates endotracheal intubation, with its attendant risks such as peri-intubation hypotension and cardiac arrest and longer-term risk of ventilator-associated complications [7].

High-flow nasal therapy delivers heated, humidified gas at high flow rates via nasal cannula. Compared to conventional low-flow oxygen, it improves comfort and tolerance, provides a small degree of positive airway pressure, reduces entrainment of room air, and may decrease the metabolic cost of conditioning inspired gas [8]. In hypoxemic respiratory failure, high-flow therapy has been associated with reduced intubation compared to conventional oxygen [9,10]. In contrast, evidence in T2RF is mixed and has focused largely on COPD, with uncertainty about the equivalence or superiority relative to NIPPV [11].

High-velocity nasal insufflation (HVNI) is a specific implementation of high-flow therapy that uses smaller-bore cannulae to deliver higher-velocity flow, generating greater nasopharyngeal pressure at comparable flow rates and enhancing washout of extrathoracic dead space [12]. Early studies suggest that HVNI may be noninferior to NIPPV for composite outcomes such as the need for intubation in mixed populations, with preliminary subgroup findings in patients with T2RF; however, these analyses are limited by design (eg, subgroup or post hoc) and predominance of COPD [13,14]. A single-prong asymmetric-flow nasal cannula design (Unicorn; Vapotherm Inc) has been proposed to facilitate carbon dioxide (CO_2_) egress and improve dead-space clearance at lower total flow rates, which may be advantageous for patients with unilateral nasal patency, facial trauma, or indwelling tubes [15]. Clinical trial data demonstrating ventilation benefits of such single-prong HVNI in T2RF are currently lacking.

While high-flow modalities are generally well tolerated, they are not without risk. Reported adverse effects include nasal dryness or irritation, epistaxis, and rare barotrauma [16,17]. Other considerations include infection control and aerosol generation, which depend on interface fit and clinical context [18,19]. Balanced against the known limitations and intolerance of NIPPV, there is a clear rationale to test whether HVNI can provide comparable ventilatory efficacy in the ED while maintaining better tolerability.

This noninferiority randomized controlled trial is designed to determine whether HVNI delivered via nasal cannula in a single-prong configuration is noninferior to NIPPV for improving ventilation in ED patients with T2RF from any cause. We hypothesize that HVNI will be noninferior to NIPPV with respect to reduction in partial pressure of CO_2_ (PaCO_2_) over an early, clinically relevant time frame, with secondary outcomes including intubation, treatment tolerance, and safety.

## Methods

### Study Design and Setting

**Figure 1.**
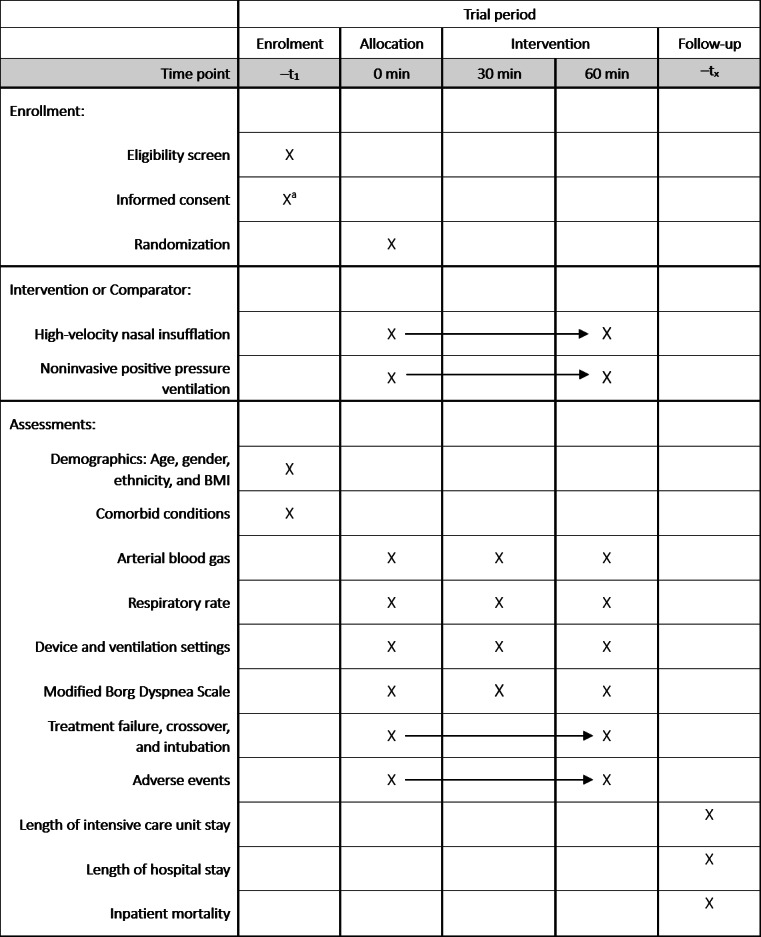
SPIRIT (Standard Protocol Items: Recommendations for Interventional Trials) 2025 schedule of enrollment, interventions, and assessments for a noninferiority randomized controlled trial. *Emergency waiver with delayed consent may be obtained from the patient or legally acceptable representative at the earliest opportunity.

This will be a single-center, noninferiority, open-label randomized controlled trial conducted in the Emergency Medicine Department of National University Hospital, Singapore, a 1225-bed tertiary academic medical center with 24/7 specialist coverage and approximately 110,000 annual attendances. Adult patients aged 21 years and above requiring NIPPV for T2RF due to any cause will be randomized to HVNI or NIPPV. The trial has been registered in ClinicalTrials.gov (NCT07065656). The protocol was prepared in accordance with the SPIRIT (Standard Protocol Items: Recommendations for Interventional Trials) 2025 statement [20]. The SPIRIT participant timeline is shown in Figure 1.

### Ethical Considerations

This study received ethics approval from the National Healthcare Group Domain Specific Review Board (reference 2024-4329) on February 24, 2025. Because patients with T2RF are deemed critically ill and hypoxia may affect their mental capacity, informed consent will be obtained from a legally acceptable representative (LAR) where feasible prior to randomization. If an LAR is unavailable and enrollment cannot be delayed, patients will be enrolled under emergency waiver, with delayed consent obtained from the LAR or the patient at the earliest opportunity prior to hospital discharge. This approach complies with Singapore’s regulations for clinical trials under emergency situations [21]. The waiver and delayed consent will be certified by a study investigator and an independent emergency medicine specialist. Individual participant data will be deidentified prior to sharing.

### Eligibility Criteria

Adult patients aged 21 years and above with T2RF, defined as arterial blood gas (ABG) showing PaCO_2_ above 45 mm Hg with pH below 7.35 due to any cause for whom ventilatory support is indicated in the ED will be included. Patients with do-not-resuscitate orders are eligible if NIPPV use is permitted. The clinical indications for initiating ventilatory support are identical for both HVNI and NIPPV in this trial as both modalities target the same pathophysiology of acute hypercapnic respiratory failure requiring augmented ventilation. The following patients will be excluded: patients with do-not-resuscitate orders that specifically do not allow for NIPPV, clinical suspicion or confirmed base of skull fracture or severe facial trauma precluding mask or cannula placement, vulnerable patient populations (eg, pregnant women or prisoners), refusal of consent (by the patient or LAR), hemodynamic instability requiring immediate resuscitation (eg, systolic blood pressure <90 mm Hg after initial fluids), cardiac or respiratory arrest, and immediate need for endotracheal intubation as judged by the attending physician. Additional exclusion criteria include contraindications specific to either modality: complete nasal obstruction or anatomical abnormalities precluding nasal cannula placement (contraindication to HVNI), recent upper-airway or esophageal surgery (contraindication to both NIPPV and HVNI), undrained pneumothorax (contraindication to positive pressure ventilation), active uncontrolled upper gastrointestinal bleeding with high aspiration risk, and inability to protect the airway (eg, excessive secretions with absent cough reflex).

### Randomization and Allocation Concealment

Enrollment will be through convenience sampling of eligible ED patients triaged as “urgent” or “immediate.” All study investigators are full-time clinicians with an average of 16 to 20 shifts per month to cover as many shifts as possible for enrollment. Participants will be randomized 1:1 to HVNI or NIPPV using variable block sizes generated by an independent web-based platform [22]. The specific block sizes are not disclosed in this manuscript to prevent prediction of the allocation sequence. Allocation will be implemented via sealed, opaque, sequentially numbered envelopes, with allocation concealment maintained until randomization is completed.

### Blinding

Due to the nature of the interventions, participants, treating clinicians, and bedside nursing staff cannot be blinded to group allocation. ABG is analyzed at the bedside using i-STAT Alinity (Abbott Laboratories). Device allocation will not be recorded on ABG stickers or forms to minimize detection bias. The data analyst performing the primary and secondary outcome analyses will also be blinded to treatment allocation and will receive deidentified datasets with treatment arms coded (eg, group A and group B) without revealing the intervention assignment. Data entry staff who are not involved in clinical care will be trained to abstract outcomes using standardized case report forms (CRFs). Nevertheless, the primary outcome evaluated is objective and should not be confounded by lack of blinding [23].

### Interventions

In the HVNI arm, patients will receive oxygen therapy through the Vapotherm high-velocity therapy 2.0 via a single-prong nasal cannula (Unicorn). Initial settings are flow rate of 35 L per minute [14], temperature of 37 °C, and fraction of inspired oxygen (FiO_2_) titrated up to 1.0 to achieve target oxygen saturation (SpO_2_). The titration algorithm will be to maintain flow rate at 35 L per minute when tolerated to optimize CO_2_ washout. If intolerance occurs (eg, agitation or discomfort), stepwise reduction in flow by 5 L per minute will be performed to the minimally tolerated level while maintaining target SpO_2_. Adjustments in temperature and FiO_2_ will be up to the attending physician’s discretion to achieve the target SpO_2_ value. Flow rate may be uptitrated as tolerated upon assessment of respiratory rate, work of breathing, and comfort. Study investigators will verify that the single-prong cannula remains securely positioned in the nostril and has not been dislodged due to patient movement. Mouth state (open vs closed) will be recorded at each assessment time point as open-mouthed breathing may reduce the effectiveness of nasopharyngeal dead-space washout. If the cannula is found to be displaced, it will be repositioned immediately, and the event will be documented. Persistent inability to maintain cannula position will be recorded as a protocol deviation.

In the NIPPV arm, patients will receive ventilation through the Respironics V60 respirator (Philips Healthcare) with an oronasal mask of appropriate size. Initial settings are IPAP of 12 to 20 cm H_2_O, EPAP of 5 to 10 cm H_2_O, and FiO_2_ of up to 1.0 to achieve the target SpO_2_. The titration algorithm will be to adjust IPAP to alleviate dyspnea and reduce respiratory rate and PaCO_2_, adjust EPAP for oxygenation and to counter intrinsic positive end-expiratory pressure when indicated, and optimize mask fit to minimize leak.

Cointerventions such as bronchodilators, systemic steroids, antibiotics, diuretics, and other standard-of-care therapies are permitted as clinically indicated and should follow departmental protocols. To reduce confounding, all cointerventions will be documented with time stamps. Initiation of major therapy changes will be avoided within 10 minutes preceding scheduled ABG draws when clinically safe.

### Oxygen Targets and Monitoring

All patients will be put on continuous pulse oximetry. For presumed acute respiratory acidosis without known chronic hypercapnia, SpO_2_ will be maintained above 95%, and for known or suspected chronic hypercapnia with acute decompensation, SpO_2_ will be kept between 88% and 92%. Each patient’s target range will be declared at baseline and maintained throughout the initial 60-minute assessment window unless clinical changes require adjustment. FiO_2_ will be titrated to maintain the declared target. Physiologic monitoring will include vital signs per ED standard, with respiratory rate recorded at baseline, 30 minutes, and 60 minutes.

### Rescue, Failure, and Crossover Criteria

The independent attending physician may change modality or escalate care if deterioration occurs. To standardize decisions, treatment failure is defined by any of the following: persistent or worsening acidosis (pH decrease ≥0.03 from baseline or pH <7.25 with respiratory rate of >30 breaths per minute after 30 minutes of therapy, PaCO_2_ increase ≥5 mm Hg at 30 minutes compared with baseline, and SpO_2_ persistently below target for >5 minutes despite FiO _2_≥0.8 [or the maximal tolerated settings]), severe intolerance to the device (eg, sustained agitation or inability to tolerate the interface) despite comfort measures, and clinical signs of impending respiratory failure (eg, decreasing consciousness, marked fatigue, and hemodynamic deterioration). Intubation criteria follow departmental standards for acute respiratory failure and will be applied consistently in both arms. All crossovers and intubations will be recorded with timing and reason.

### Outcome Measures

The primary outcome is the percentage change in PaCO_2_ from baseline to 60 minutes after initiation of therapy. Baseline is defined as the pretreatment ABG obtained before initiation of the assigned therapy (HVNI or NIPPV). This ensures that the primary outcome reflects the ventilatory effect of the intervention itself. The 30- and 60-minute assessment time points are measured from the time of therapy initiation. The ABG results and other variables (Table 1) to be collected will be documented in real time on paper-based CRFs by trained study investigators.

**Table 1.** Summary of recorded patient variables in a noninferiority randomized controlled trial comparing high-velocity nasal insufflation (HVNI) vs noninvasive positive pressure ventilation (NIPPV) for type 2 respiratory failure in adult emergency department patients at National University Hospital, Singapore.

Variables	Measure
Primary outcome measure	Change in PaCO_2_^a^ at 60 min obtained after initiation of HVNI or NIPPV compared to baseline (before initiation of therapy)
Secondary outcome measures	Change in PaCO_2_ at 30 min compared to baseline Changes in respiratory rate 30 and 60 min after initiation of HVNI or NIPPV Modified Borg Dyspnea Scale at 30 and 60 min Treatment failure Conversion to another modality of oxygenation Requirement for intubation in the emergency department Adverse events due to therapy
Demographics	Age Gender Ethnicity BMI
Comorbidities	Congestive cardiac failure Ischemic heart disease Atrial fibrillation Chronic obstructive pulmonary disease Asthma Obstructive sleep apnea Diabetes mellitus Bronchiectasis Restrictive lung disease Neuromuscular disease Opioid or benzodiazepine use
Vital signs	Temperature Heart rate Respiratory rate Systolic and diastolic blood pressure Mean arterial pressure Glasgow Coma Scale
Investigations	Chest x-ray findings Arterial blood gas results at 0, 30, and 60 min
Clinical course and secondary clinical outcomes	Emergency department diagnosis Hospital inpatient discharge diagnosis Length of intensive care unit stay (if any) Length of hospital stay Inpatient mortality

a PaCO_2_: partial pressure of carbon dioxide.

Percentage change (relative difference) as the primary outcome was selected to account for the wide range of baseline PaCO_2_ values expected in a heterogeneous, all-cause T2RF population. An absolute change in PaCO_2_ may disproportionately favor patients with higher baseline values as they have greater room for absolute reduction. The percentage change normalizes the treatment effect relative to each patient’s starting value, facilitating more equitable comparison across the spectrum of disease severity. To address the potential limitation of relative outcomes, a prespecified sensitivity analysis using absolute change in PaCO_2_ at 60 minutes will be conducted to confirm the robustness of the primary finding.

Secondary outcomes include the following: percentage change in PaCO_2_ from baseline to 30 minutes; change in respiratory rate at 30 and 60 minutes; modified Borg Dyspnea Scale (0-10) at 30 and 60 minutes [24]; proportion of treatment failure (as defined above), conversion to another modality of oxygenation, and need for intubation in the ED; adverse events due to therapy rendered (eg, facial pressure injury, vomiting, or aspiration); length of intensive care unit (ICU) and hospital stay; and inpatient mortality. Inpatient mortality will be assessed at hospital discharge. ICU length of stay (if applicable) and total hospital length of stay will be recorded in days from the date of ED presentation to the date of ICU discharge and hospital discharge, respectively. These data will be abstracted from the electronic health record (EHR) following patient discharge.

### Sample Size

A total of 84 patients (42 per group) will be enrolled to provide 80% power and one-sided α of 2.5% to demonstrate noninferiority [25] assuming an SD of 6.65% for percentage change in PaCO_2_ levels before and after treatment [26] and a noninferiority margin of 4.3%. The sample size includes an estimated 10% allowance for missing primary end point data. The noninferiority margin of a 4.3% absolute difference in percentage change was informed by clinician judgment (number of specialist accredited [board-certified] emergency physicians, n=14) of clinically acceptable differences and literature on PaCO_2_ responses to NIPPV. For example, emergency physicians opined that a change from 70 mm Hg to 63 mm Hg using HVNI (−10.0%) was considered noninferior to a change in PaCO_2_ from 70 mm Hg to 60 mm Hg after treatment with NIPPV (−14.3%).

### Statistical Analysis Overview

The primary analysis will follow an intention-to-treat approach, with supportive per-protocol analysis excluding major deviations (eg, early crossovers or missing postbaseline ABG values) and a safety set analyzed as treated. The main end point, percentage change in PaCO_2_ at 60 minutes, will be assessed using analysis of covariance (ANCOVA) adjusting for baseline PaCO_2_ and prespecified covariates (eg, etiology, baseline pH, and age). Noninferiority will be concluded if the upper bound of the 2-sided 95% CI for the HVNI–NIPPV adjusted difference is below the 4.3% margin. A prespecified sensitivity analysis will repeat the primary ANCOVA model using absolute change in PaCO_2_ at 60 minutes as the dependent variable to assess whether the conclusions are robust to the choice of relative vs absolute outcome metric. Secondary endpoints (percentage PaCO_2_ changes at 30 minutes, respiratory rate, and modified Borg Dyspnea Scale) will use similar adjusted models, whereas binary outcomes (treatment failure, intubation, and adverse events) will be compared using chi-square or Fisher exact tests and logistic regression, and length of stay and mortality will be assessed via nonparametric or time-to-event and logistic methods. Missing data handling will focus primarily on the primary outcome (percentage change in PaCO_2_ at 60 minutes). Missing 60-minute ABG results may occur due to early treatment crossover, intubation before the 60-minute time point, patient refusal of repeat ABG, or technical failure of the ABG analyzer. Multiple imputation by chained equations will be performed, with the imputation model including baseline PaCO_2_, baseline pH, treatment arm, age, etiology, respiratory rate, and 30-minute PaCO_2_ (if available). A minimum of 20 imputed datasets will be generated, and results will be pooled using the Rubin rules.

Sensitivity analyses will include (1) complete-case analysis (excluding participants with missing primary outcome data) and (2) worst-case imputation for the HVNI arm (assigning the worst observed percentage change to missing values in the HVNI arm and the best observed percentage change to missing values in the NIPPV arm), which is a conservative approach for noninferiority trials. Baseline covariates with missing data (expected to be minimal) will be handled through multiple imputation within the same multiple imputation by chained equations framework. Missing secondary outcomes will be analyzed using available data, with the extent of missingness reported for each outcome. Secondary endpoints will be treated as exploratory without formal multiplicity adjustment.

Prespecified exploratory subgroup analyses will be conducted to assess potential heterogeneity of treatment effect. Subgroups will include (1) COPD vs non-COPD etiology, (2) baseline PaCO_2_ strata (eg, 45‐60 mm Hg vs >60 mm Hg), and (3) baseline pH strata (eg, 7.25‐7.35 vs <7.25). Subgroup effects will be evaluated by including interaction terms (subgroup × treatment) in the primary ANCOVA model. These analyses are exploratory and hypothesis generating; the trial is not powered for subgroup comparisons, and results will be interpreted with caution.

### Data Collection and Management

Data will be recorded on paper-based standardized CRFs using trial numbers only and will be stored under lock and key with restricted access. Electronic data will be stored on secure, access-controlled systems with password protection and audit trails. The ABG samples will be analyzed at the bedside using the i-STAT Alinity analyzer. The analyzer generates a printed result slip that includes PaCO_2_, PaO_2_, pH, bicarbonate, and other parameters with a time stamp. The printed result slip will be attached to the paper-based CRF for each participant. Should the printed result slip be unavailable, illegible, or degraded over time, the ABG results will be retrieved electronically from the analyzer and from the patient’s EHR. Study investigators will transcribe key ABG values onto the CRF in real time. Device settings (flow rate, FiO_2_, and temperature for HVNI and IPAP, EPAP, and FiO_2_ for NIPPV) will also be recorded on the CRF at baseline, 30 minutes, and 60 minutes. Accordingly, the study CRF serves as the primary source document to ensure complete and accurate capture of primary outcome data.

Apart from ABG readings, other variables and outcomes as stated will be collected and followed up on through electronic medical record review. To ensure data accuracy, the following quality checks are in place: (1) double data entry by 2 independent data entry staff members, with discrepancy resolution; (2) programmed range and logic checks during electronic data entry; (3) periodic source data verification by the study coordinator comparing CRF entries against ABG printouts and EHR records; and (4) audit trails for all electronic data modifications.

### Quality Assurance and Monitoring

Study investigators will undergo training on device application, titration protocols, mask fitting and leak management, and CRF completion. An independent data monitoring and safety team within the institution will conduct periodic reviews to assess protocol adherence, data completeness, and safety. Any serious adverse events related to the study interventions will be reported to the National Healthcare Group Domain Specific Review Board per institutional requirements. Quarterly adherence checks will include verification of device settings, oxygen targets, ABG timing windows, and documentation of cointerventions.

## Results

The trial was prospectively registered in ClinicalTrials.gov (NCT07065656) on July 15, 2025. Recruitment commenced in January 2026 at the Emergency Medicine Department of National University Hospital, Singapore. As of July 2026, a total of 19 participants have been enrolled out of a target sample of 84. Recruitment is expected to be completed by June 2027. Data analysis will commence following completion of enrollment, with results expected to be published by 2028.

**Figure 2.**
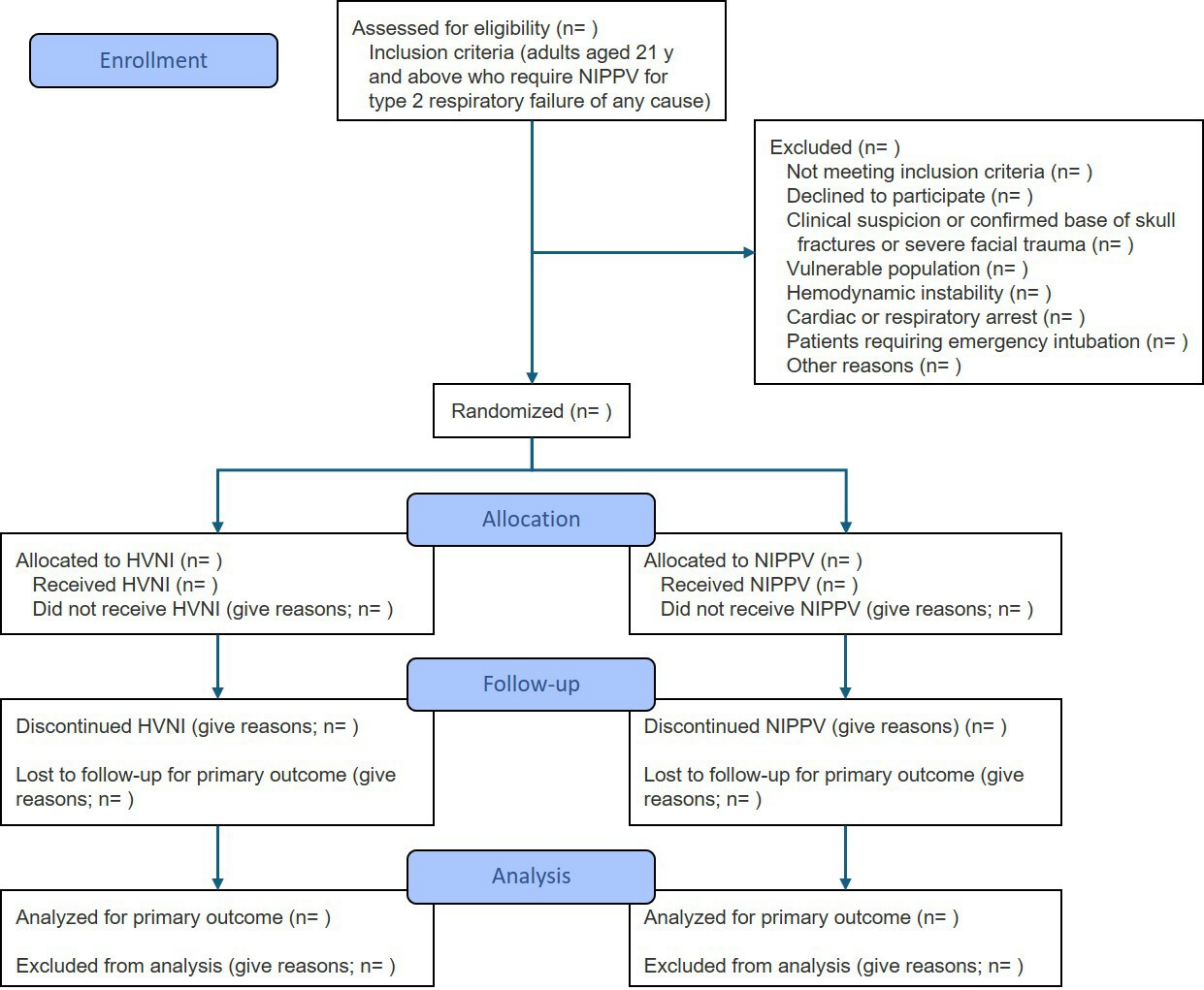
CONSORT (Consolidated Standards of Reporting Trials) 2025 flow diagram for a noninferiority randomized controlled trial comparing high-velocity nasal insufflation (HVNI) vs noninvasive positive pressure ventilation (NIPPV) for type 2 respiratory failure in adult emergency department patients at National University Hospital, Singapore.

The completed trial will be reported according to the CONSORT (Consolidated Standards of Reporting Trials) 2025 statement and its extension for noninferiority trials [27,28]. The planned CONSORT flow diagram is illustrated in Figure 2.

## Discussion

At comparable oxygen flow settings, HVNI may generate higher airway velocities than conventional high-flow nasal oxygenation, potentially improving ventilatory support while using similar oxygen flow. These characteristics could translate into operational and sustainability advantages if supported by measured reductions in cumulative oxygen consumption and resource use in clinical practice. In this trial, oxygen use and related resource metrics will be measured to substantiate any such claims.

If HVNI is found to be noninferior to NIPPV, it could offer a practical alternative in scenarios where mask-based interfaces are poorly tolerated or contraindicated. Potential advantages include fewer mask-related skin injuries, improved ability to communicate and take oral medications, and easier access for airway toileting and suctioning. These hypotheses will be evaluated alongside safety outcomes, recognizing that HVNI can have its own adverse effects (eg, nasal discomfort, mucosal dryness, and epistaxis) and that aspiration risk requires vigilance despite facilitated suctioning. Infection prevention measures appropriate for high-flow therapies will be followed in accordance with institutional policies.

In this study, HVNI will be delivered via a single-prong cannula (Unicorn) as a novel treatment for T2RF from all causes. We hypothesize that, through generation of nasopharyngeal pressure and high-velocity flow, HVNI may enhance extrathoracic dead-space washout and reduce PaCO_2_, achieving ventilatory effectiveness that is noninferior to NIPPV in acute T2RF. Device setup, flow titration, and monitoring will be standardized, with staff training to promote consistent delivery and protocol fidelity.

This trial builds on existing work in several ways. A prior study on single-prong nasal oxygenation focused largely on symptom relief in patients with COPD rather than ventilatory effectiveness in T2RF [15]. Randomized comparisons of HVNI and NIPPV in T2RF have predominantly enrolled COPD populations [11]. By evaluating ventilatory outcomes via a single-prong interface and enrolling patients with T2RF from any cause, this study addresses gaps in the literature and reflects the diagnostic heterogeneity commonly encountered in emergency care.

Exploratory subgroup analyses (eg, COPD vs non-COPD and baseline PaCO_2_ strata) are prespecified and described in the Statistical Analysis Overview section. These analyses are designed to assess potential heterogeneity of treatment effect across clinically relevant subpopulations, recognizing that the trial is not powered for formal subgroup comparisons.

A noninferiority design is appropriate given that NIPPV remains the standard of care for acute T2RF and a more tolerable, operationally streamlined alternative could be clinically valuable. The noninferiority margin of the primary end point (change in PaCO_2_ at a prespecified early time point of 60 minutes) is defined a priori and justified on clinical grounds informed by the expected magnitude of PaCO_2_ improvement with NIPPV in similar populations. Nevertheless, noninferiority trials are inherently vulnerable to bias toward equivalence as factors that dilute true treatment difference, such as protocol deviations and nonadherence, tend to favor noninferiority by minimizing observed differences between interventions [28]. The study protocol seeks to mitigate this risk by incorporating complementary analyses using both intention-to-treat and per-protocol approaches to ensure valid interpretation of noninferiority findings. Predefined rescue criteria for escalation and intubation will be used to reduce performance bias in clinician-driven decisions.

This single-center trial design supports consistent implementation but may limit generalizability to settings with different resources or experience with HVNI. Blinding of patients, health care providers, and research staff is not feasible and may influence subjective measures and escalation decisions. These risks are mitigated by an objective primary end point, standardized timing of ABG sampling, predefined escalation criteria, and complementary intention-to-treat and per-protocol analyses. In addition, while PaCO_2_ levels at 30 and 60 minutes provide a clinically relevant and objective indicator of early ventilatory response, PaCO_2_ is a surrogate outcome of short-term response and does not fully capture longer-term ventilation success or failure. Physiologically, reduction in PaCO_2_ levels may be related to increased minute ventilation brought about by dyspnea rather than true clinical improvement. Patients with T2RF from COPD and neuromuscular diseases often follow a progressive disease trajectory and require extended ventilatory support to prevent delayed clinical deterioration, and taking PaCO_2_ measurements in the early treatment window may fail to reflect longer-term clinical effectiveness of the intervention [29]. Finally, the use of percentage change in PaCO_2_ as the primary outcome, while it accounts for baseline severity differences, may introduce variability in treatment effect estimation across patients with heterogeneous baseline values. The relationship between PaCO_2_ and alveolar ventilation is nonlinear such that equivalent percentage changes may represent different physiological magnitudes at different baseline levels.

Using early change in PaCO_2_ as the primary outcome represents both a strength and a limitation. PaCO_2_ directly reflects alveolar ventilation and offers an objective, timely measure of ventilatory response. However, it is a surrogate that may not fully capture longer-term clinical effectiveness, and its relationship to ventilation is nonlinear across baseline values; equivalent absolute changes can imply different physiological effects at different baseline PaCO_2_ levels [30]. Early reductions may overestimate clinical benefit in some patients, particularly those presenting with severe hypercapnia and very high baseline PaCO_2_ levels. Accordingly, interpretation of the primary end point will be integrated with prespecified secondary outcomes and sensitivity analyses. If noninferiority is established with acceptable safety and tolerance, subsequent clinical trials focused on patient-centered and longer-term outcomes, including cost-effectiveness and broader implementation, would be warranted.

## Abbreviations

ABG: arterial blood gas
ANCOVA: analysis of covariance
CO_2_: carbon dioxide
CONSORT: Consolidated Standards of Reporting Trials
COPD: chronic obstructive pulmonary disease
CRF: case report form
ED: emergency department
EHR: electronic health record
EPAP: expiratory positive airway pressure
FiO_2_: fraction of inspired oxygen
HVNI: high-velocity nasal insufflation
ICU: intensive care unit
IPAP: inspiratory positive airway pressure
LAR: legally acceptable representative
NIPPV: noninvasive positive pressure ventilation
PaCO_2_: partial pressure of carbon dioxide
SPIRIT: Standard Protocol Items: Recommendations for Interventional Trials
SpO_2_: oxygen saturation
T2RF: type 2 respiratory failure
